# Nutrition Education Needs and Barriers of Uninsured Clients who Utilize Free Clinics in Western North Carolina

**DOI:** 10.13023/jah.0603.04

**Published:** 2024-10-01

**Authors:** Manan Roy, Alisha Farris, Erin Loy, Lauren Sastre, Danielle L. Nunnery

**Affiliations:** Appalachian State University; Appalachian State; Appalachian State University; East Carolina University; Appalachian State University

**Keywords:** Appalachia, education, free clinic, health care, medically uninsured, nutrition

## Abstract

**Introduction:**

Many uninsured adults rely on free health clinics for prevention and treatment of chronic disease. Little is known about the nutrition education needs of adults served by free health clinics, especially those living in counties within the Western North Carolina Appalachian Mountain Region.

**Methods:**

An in-person survey was distributed to 202 clients of two free health clinics in western North Carolina. Descriptive analyses were conducted to determine frequency distributions for food and physical activity practices, acceptable topics and strategies for nutrition education, and the acceptance and barriers for various modalities.

**Results:**

Depending on the clinic, 49–58% of participants were female with an average age of 45, and Caucasian (48–66%). Around half reported barriers to cooking. The majority frequently ate takeout and engaged in exercise. Participants were most interested in receiving local produce and recipes and were most likely to use a smartphone for nutrition information. Participants preferred actionable interventions but needed help overcoming barriers to food access and cooking.

**Implications:**

Future interventions within clinics should focus on assessing patient needs and tailoring services. As transportation was the most commonly cited barrier, clinics could leverage online modalities to enhance clinic education in this population since a majority of clients had access to the internet via smartphone and over half cited interest in online nutrition education.

## INTRODUCTION

More than 1 million people in North Carolina (NC), or roughly 11% of the U.S. population, are uninsured.^1^ The Affordable Care Act expanded Medicaid to include adults under age 65 who earn up to 138 percent of the federal poverty level, and working families earning between 100 and 400% of it.^2^ However, not all states have adopted this expansion — NC included. Due to the lack of Medicaid expansion, many individuals are left in a healthcare “gap,” in which they do not qualify for Medicaid due to their income level, yet still cannot afford private insurance on their own.^3^ This gap in insurance coverage leaves many people without access to health care.

Free health clinics help bridge this gap for many in NC and across the U.S., providing essential services regardless of an individual’s ability to pay.^4^ A free health clinic is defined as a private, non-profit, community-based organization that provides free or greatly reduced-cost medical care to low-income, uninsured, or underinsured persons. Often these clinics utilize volunteer healthcare professionals and partner with other health providers to provide critical services.^5^ According to the National Association of Free & Charitable Clinics (NAFC), there are over 1,400 free clinics nationwide^6^ and 88 clinic sites in NC as of 2022.^7^ They are safety-net healthcare organizations which are private and non-profit, and utilize a volunteer model to provide medical, dental, pharmacy, vision, and/or behavioral health services.^5^,^6^ They target low-income, uninsured, and underinsured adults and have no fee or include a sliding fee scale based on income.^5^ Additionally, free clinics are typically community based, allowing the clinic to tailor to each unique community.^5^ These free clinics are distinct from government funded Federally Qualified Health Centers (FQHC). Both free clinics and FQHCs provide health care to an underserved population, serving an estimated two million patients nationwide each year, with 189 FQHCs located in NC.^6^ FQHCs receive funding from the government and are more likely to be able to provide more services and employ a larger staff. For free clinics without FQHC designation, limited funding, staff, and time may hinder their ability to provide nutrition-specific services that could greatly impact the health of their clients.^6^

Food insecurity is strongly correlated with chronic diseases such as diabetes and hypertension.^8^ Rural, low-income, and minority populations are often at an increased risk of chronic diseases and food insecurity, and they are disproportionately affected by lack of resources and lack of access to healthcare services.^8^,^9^ A lack of access to healthcare services and potential delay of diagnosis can exacerbate chronic disease,^10^ leading to worse health outcomes. In two studies with uninsured adults, most of whom identified as women and Latino/Hispanic, one study found 55% of participants had at least one chronic disease, and the other study found that a majority (74%) of free health clinic clients were food insecure.^10^,^11^

Since many chronic diseases are manageable or preventable with dietary modifications,^12^ nutrition education and access to chronic disease management is critical in addressing food insecurity and impacting the food security status of households.^13^ When available, nutrition education programs have demonstrated their effectiveness at improving participants’ knowledge of nutrition, eating behaviors, and health.^14^ However, nutrition education cannot be effective if participants do not have access to healthy food and the support of their environment and healthcare system to make healthy choices.^15^,^16^ There remains limited knowledge about the nutrition education needs and potential intervention strategies for uninsured adults who must rely on free health clinics.^11^ Additionally, those living in Appalachia, including the Western part of NC, are disproportionately impacted by food insecurity, diet-related chronic diseases, and overall lack of access to health care, often due to geographic isolation from resources.^17^–^18^

It is critical to understand the unique barriers that uninsured adults experience living in the Western NC region of Appalachia. Most importantly, understanding these challenges from the perspective of the patients themselves could ensure that patients have a clear voice and ownership in health care decisions made at their free clinics. Patient engagement in their healthcare decisions ultimately improves health behaviors and leads to better overall care.^19^ The aim of this study was to evaluate the current food-shopping, cooking, and physical activity practices of uninsured adults accessing free clinics; acceptable topics and strategies for providing nutrition education to uninsured adults; and the feasibility and acceptance of various nutrition education modalities. To answer these questions, an exploratory needs assessment survey of participants in two free health clinics (without FQHC designation) in Western NC was conducted.

## METHODS

### Participants and Setting

**Figure 1:**
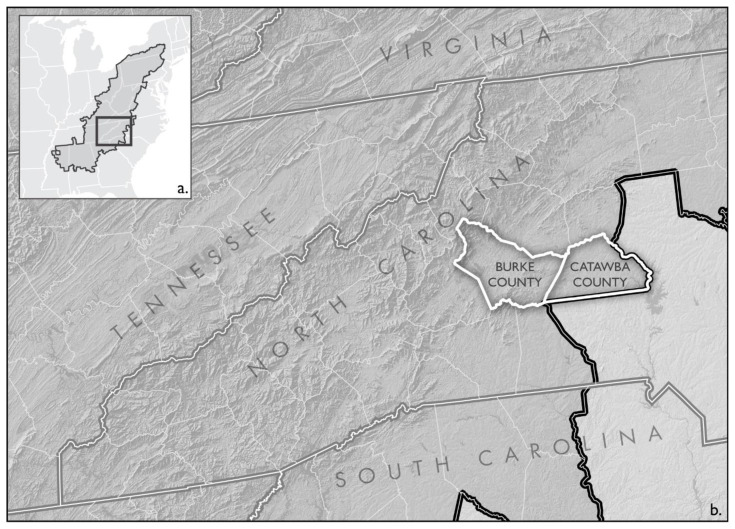
NOTE: (a) Locator map of Appalachian Regional Commission (ARC) boundary (black outline). (b) Burke and Catawba counties highlighted to denote counties for each clinic included in the study. Both counties fall within the ARC boundary.

This exploratory needs assessment took place in a community-based setting in two distinct counties in the Appalachian Region of Western NC (Catawba, and Burke – see Fig. 1) through a partnership with two free health care clinics (without FQHC designation). These clinics are anonymized and denoted as “DDN” and “HTD.” In addition to free primary health care, DDN is a faith-based organization whose goal is to assist individuals in gaining independence from poverty. It provides financial aid to prevent eviction or utility disconnection, laundry services, access to a housing specialist, access to showers, a voucher for free clothing, and a food pantry. The DDN clinic offers laboratory services, referrals, pharmacy and dental services. The HTD clinic is also a faith-based organization providing free health care, pharmacy services, a thrift store, food pantry, referrals, dental, and laboratory services. The HTD clinic additionally has a Farm Worker Health Program specific to migrant and seasonal workers. This clinic provides transportation to healthcare appointments, access to primary physicians, dental services, telehealth for behavioral health, and laboratory services, and often provides care at the workers’ field site. While client demographics and many of the services vary between locations, they both offer doctor visits on an income-based sliding scale for those denied by Medicaid. Individuals who attended one of the above clinics and were aged 18 years or older were recruited to participate in an anonymous survey. Participants were invited to take the survey in waiting rooms and/or clinic rooms of the facility while waiting to be seen by the clinic staff. Surveys could be completed individually or read aloud by a research assistant. Surveys were collected in a 4-month period, from November 2019 to early March 2020. This study was considered exempt by the Appalachian State University Institutional Review Board and approved for distribution by clinic directors.

### Survey Design

The anonymous questionnaire consisted of 65 questions (i.e., 58 closed-ended items and seven open-ended 7 items to elaborate on closed-ended responses) and took an average of approximately 10 minutes to complete. The survey was loaded onto tablet devices using the REDCap program through the university and administered by graduate research assistants. Questions were focused on current food-shopping, cooking, and physical activity practices, acceptable topics and strategies for providing nutrition education, the feasibility and acceptance of various nutrition and activity education modalities (that clinics could offer or were considering offering), and patient demographics. The survey was developed in coordination with the free clinics. Literacy level, content validity, and face validity were evaluated independently by three nutrition professors familiar with participatory research, food access, and nutrition education literature. The survey was translated from English to Spanish through a third-party interpreter, then verified and corrected for content and fidelity of meaning with a second translator. Graduate research assistants and clinic staff were also available to read, translate, and interpret the survey if the participant needed assistance for low literacy or needed translation from formal Spanish to conversational Spanish.

### Food Shopping, Cooking, and Physical Activity

This survey section focused on food shopping and cooking, physical activity behaviors, barriers to cooking and physical activity, and desired topics of information in an open-response or “yes/no” question format. For cooking, one example question was, “Is there anything that stops you from cooking? If so, what stops you?”. For physical activity, example questions were, “How many times a week do you exercise?”, “Do you have anything that prevents you from exercising?”, and “What kind of information would be helpful to you in learning about physical activity?”.

### Topics and Strategies for Nutrition and Physical Activity Education

This section focused on nutrition/physical activity interventions participants would be interested in at their clinic that were being considered by clinic administration. Participants indicated their interest by responding to various topic and education strategies with Likert scale responses of “yes, very;” “yes, somewhat;” “neutral;” “no;” “not sure;” or “N/A.” Participants were first asked about six different strategies: (1) receiving local produce; (2) recipe ideas for produce; (3) taste or cooking workshops; (4) home garden support; (5) grocery store tours; and (6) home visits by a nutritionist. Participants were then asked about their interest in seeing a nutritionist to develop a food plan and/or working with a personal trainer to develop a physical activity plan for: overall health, weight management, diabetes, blood pressure, and heart disease. This was followed by an open-response option to capture interests that were not asked about in the survey.

### Education Modalities

This section focused on technology use and potential modalities for providing nutrition and physical activity education and used yes/no questions. Example questions were, “Do you have a smartphone?”, “Do you have regular internet access?”, and “Do you use social media?”. Additionally, participants responded yes/no to their interest in receiving health, nutrition, or physical activity education through social media or online videos.

### Sociodemographics

The questionnaire concluded by eliciting information about the descriptive characteristics of participants, including age, gender, race, ethnicity, education, employment status, transportation, household members, and food assistance participation. These were assessed through a mix of closed- and open-ended questions.

### Data Analysis

Descriptive statistics were conducted with Stata 15 statistical software (StataCorp. 2017. Stata Statistical Software: Release 15. College Station TX: StataCorp LLC.). For nutrition education questions, ordinal variables were divided across participants’ interest level as follows: 1= “Yes, Very much;” 2 = “Yes, somewhat;” 3 = “Neutral;” 4 = “No;” 5 = “Not Sure;” and 6 = “N/A.” These categories were then collapsed into a binary variable with a value of 1 for “Yes, very much” or “Yes, somewhat,” and zero otherwise. For the variable on food label use at time of purchase, an affirmative response of “Yes, most of the time” or “Yes, sometimes” was combined into one category and given a value of 1, and negative responses were coded into a second category as zero.

## RESULTS

Overall, 202 surveys were collected, 114 from DDN (56%) and 88 from HTD (44%). Table 1 shows that more women responded to the surveys across both sites compared to men (49% v. 40% at DDN and 58% v. 32% at HTD). The participants were almost similar in age (mean = 45 ± 12.8) across the sites. DDN participants were predominantly Caucasian (67%) while 48% of HTD participants were Caucasian. About 18% of DDN participants were black, while only about 6% of HTD identified as black. Almost 47% of HTD participants identified as Hispanic, in a sharp contrast to only 4% of DDN participants.

Unemployment rates were high, with close to 60% of the DDN participants unemployed compared to about 42% of the HTD participants. Household size was comparable across the two sites and among those who responded, more DDN participants utilized the SNAP program (36% v. 25%) and utilized a food pantry (36% v. only 13%). Both sites had low WIC participation with 4% at DDN and 6% at HTD.

### Food shopping, cooking, and physical activity

About 61% of DDN participants and 45% of HTD participants reported at least one issue that prevented them from cooking. Almost 65% of DDN participants reported eating at restaurants along with 82% of HTD participants. DDN clients reported eating at restaurants an average of two days per week, while HTD clients reported eating at restaurants an average of once per week. About 62% of DDN clients reported looking at food labels most of the time or sometimes during food purchasing compared to only about 39% of HTD clients. Similarly, a majority (71%) of DDN clients were very confident or somewhat confident about food labels compared to about 44% of HTD clients. The DDN participants reported walking almost five days a week on average while HTD participants reported walking close to three days per week on average. HTD participants were more likely to do weight training than their DDN counterparts (26% v. 12.5%). HTD participants also showed greater interest in physical activity than the DDN participants (54% v. 27%). Both groups of participants were almost equally likely to mention at least one barrier to physical activity (47% for DDN participants v. 44% for HTD participants). Commonly cited barriers included time, space, and physical health limitations (i.e., pain or mobility issues).

### Topics and Strategies for Nutrition and Physical Activity Education

Table 2 reports findings from the needs assessment of the clinic clients in terms of transportation access, topics of interest and strategies for education, and interest in nutrition education modalities. More HTD participants had a personal vehicle for transportation (55% v. 40% for DDN) and relied more on friends and family for transportation as well (27% v. only 7%).

In both clinics, participants were overwhelmingly interested in receiving local produce (79% in DDN and 85% in HTD) (Table 2). The second-most-popular interest was in receiving recipes (around 61% in DDN and 84% in HTD). A greater proportion of HTD participants were interested in all but one nutrition education strategy — only 47% of HTD participants expressed interest in test tasting/cooking class compared to 61% of DDN participants. Both site participants expressed the least interest in home visits by a nutritionist (about 23% of DDN and about 31% of HTD).

For both clinics, participants expressed the most interest in a nutrition education plan on overall health (34% for DDN; 41% for HTD). An education plan on blood pressure management was the second highest of interest for the DDN participants, and weight management represented the second highest interest for the HTD participants. The DDN participants were least interested in a food plan for diabetes and heart disease while HTD participants were least interested in food plans for heart disease and blood pressure (Table 2).

For both clinics, participants expressed the most interest in a physical activity education plan to support overall health (33% for DDN; 42% for HTD) and weight management (28% for DDN; 38% for HTD). The DDN participants were least interested in a physical activity plan focused on diabetes and heart disease management while HTD participants were least interested in a physical activity plan for heart disease and blood pressure (Table 2).

### Online Access and Interest in Nutrition Education Modalities

Just over half of participants at each clinic reported owning a smartphone (DDN (56%) and HTD (64%), and over two-thirds reported participants reported using their smartphone to look up nutrition information (i.e., Google) DDN (66%) and HTD (81%). However, only about 25% had a data plan on average, and about 12% reported that this affected their internet or text use and availability. Both groups of participants used social media roughly at the same rate (54% for DDN and about 53% for HTD). The difference in internet use across the clinics showed greater internet access for HTD clients than for DDN clients (63% versus about 41%). About one-third of participants expressed interest in receiving nutrition education through social media (28% at DDN and 33% at HTD) (Table 2).

## CONCLUSIONS AND IMPLICATIONS

This needs assessment highlights the health practices of uninsured adults in Western NC accessing free clinics, the desired education and potential program needs, and feasibility and acceptance of various nutrition education modalities and strategies, adding to the limited literature on these concepts among the uninsured. Nutrition services such as receiving local produce and recipe ideas were more desired (79% and 85%, respectively) than nutrition or physical activity education, and participants reported high confidence in using food labels. This suggests participants in this study were aware of the benefits of local produce consumption and ready to take action but needed help overcoming barriers to access and cooking. While both clinic sites in this study had an available food pantry, participants also desired more fresh food items via options like produce boxes (Table 2). In a study by Izumi et al, clients of FQHCs who were provided produce boxes had increased variety and consumption of vegetables and an overall improved diet quality.^20^ Produce boxes provided by clinics or fruit and vegetable prescription programs are innovative strategies that could address this identified need. Fruit and vegetable prescription programs are typically clinic-based and utilize provider-generated “prescriptions” for locally grown foods. These programs have demonstrated positive improvements in produce intake and access among low-income clinic populations.^21^,^22^

In addition to produce access, participants in this study also reported barriers to cooking and frequently eating outside of the home. These findings are consistent with previous research highlighting the complexity of food access. For example, previous studies have reported that high food prices and lack of time for cooking are primary barriers for low-income individuals.^23^,^24^ Other studies have reported more concrete barriers such as lack of equipment and limited transportation to a grocery store, often complicated by living in areas considered to be food deserts. 23–25 Food deserts are defined as living >10 miles from a supermarket in rural areas or >1 mile in urban or micropolitan areas and are common in Appalachia, affecting an estimated 2.3 million Americans. 26 The present study’s findings suggest clinic services provided by nutritionists should include not just recipes, but budget-friendly, time-saving recipes and the provision of cooking equipment resources to meet client needs.

Social support has been documented as an important aspect for the success of education programs.^20^ Around half of participants were interested in tips or support through social media and/or online videos, and of those, most were utilizing Facebook. Online modalities such as Facebook could be a potential source of social support for clinics serving uninsured populations, and especially for education outreach. Social media provides a low-cost avenue for interacting with the community and providing information.^27^, ^28^ The data for this study were collected prior to the coronavirus pandemic of 2019, which has increased use and acceptability of technology for all ages and income levels.^29^ Since around half of participants were interested in education via technology prior to the pandemic, this education strategy should be explored further now that participants are likely more familiar with receiving information in this avenue. More research is needed on the feasibility and success of online nutrition education for uninsured adults who rely on free health clinics.

This study highlights the need to conduct critical needs assessments among free health clinics and the importance of tailoring intervention resources to the unique needs of that clinic and region. While both clinics are located in semi-rural western counties of NC, both clienteles had varying interests and barriers. Additionally, while there were overarching themes discussed above, several differences should be acknowledged. For example, unemployment was higher for DDN clients, likely leading to their increased use of the clinic food pantry and reported lack of cooking resources, while clients at the HTD clinic reported more barriers to transportation but more interest in physical activity. These combined overarching themes and differences should be incorporated to create appropriately tailored education for each clinic. For education/interventions to be sustainable, partnerships between the clinics and experts of nutrition and physical activity should continue to be explored and leveraged. Needs assessments such as this can assist clinics in understanding their population’s assets and gaps to achieve healthy lifestyles, as well as ensure effective grant targeting and allocation of funding for programs.^4^, ^22^, ^25^

Strengths of this study were the ability to partner with free health clinics and provide needs assessment results to stakeholders, alongside the opportunity to investigate specific client interests and barriers to create tailored education. The survey was created using appropriate reading-level language and health literacy levels for the target population including the support of an interview team and bilingual clinic staff who could assist in completing the survey. Limitations, however, were that this study cannot be generalized for all clients of free health clinics. Every clientele base has different needs and barriers that should individually be assessed. This study only serves to highlight an underserved free clinic population and provide a basic framework for how other clinics might pursue a nutrition focused needs assessment. Due to the impact of the coronavirus pandemic and timing of data collection, uninsured clients may face new challenges and barriers that were not captured with this survey. Future research should seek to compare the needs of the uninsured before and after the pandemic to understand new challenges, needs and opportunities to support free clinic clients.

**SUMMARY BOX**

**What is already known about this topic?**

Free clinics are a critical interface of primary care and chronic disease management for many vulnerable populations who fall into the healthcare coverage gap. These clinics often serve as a critical bridge to improve food access by supporting patients with food pantry items, nutrition education, and access to prevention services.

**What is added by this report?**

There is a lack of research highlighting the unique interests, needs, and barriers of patients who receive nutrition education and services through free clinics. The findings of this study suggest that clients face considerable transportation barriers. However, many clients report having access to the internet via smartphones and would be interested in more clinic engagement in nutrition education through online options like social media.

**What are the implications for future research?**

Research examining the specific and unique needs and barriers of free clinic clients can ensure that findings are used to better inform funding models and program development that is tailored and effective.

## Figures and Tables

**Table 1:** Characteristics of Entire Sample (N=202) and Comparisons of Clients from the Two Free Health Clinics

	Total Sample	DDN (n=114)	HTD (n=88)
Participant Characteristics			
	N (%)	N (%)	N (%)
**Gender**			
Male	74 (36.6)	46 (40.4)	28 (31.8)
Female	107 (53.0)	56 (49.1)	51 (58)
Not Reported	20 (9.9)	12 (10.5)	8 (9.1)
Prefer not to say	1 (0.5)	0 (0.0)	1 (1.1)
**Race/Ethnicity**			
Black	26 (12.9)	21 (18.4)	5 (5.7)
Caucasian	118 (58.4)	76 (66.7)	42 (47.7)
Asian	2 (1.0)	1 (0.9)	1 (1.1)
American Indian/Alaska Native	2 (1.0)	2 (1.8)	0 (0.0)
Not Reported	54 (26.7)	14 (12.3)	40 (45.5)
**Ethnicity**			
Hispanic	46 (22.8)	5 (4.4)	41 (46.6)
Non-Hispanic	109 (54.0)	72 (63.2)	37 (42.1)
Prefer not to say	2 (1.0)	2 (1.8)	0 (0.0)
Not Reported	45 (22.3)	35 (30.7)	10 (11.4)
**Employment status**			
Unemployed	105 (52.0)	68 (59.7)	37 (42.1)
Full time	36 (17.8)	11 (9.7)	25 (28.4)
Part time	28 (13.9)	15 (13.2)	13 (14.8)
Not Reported	33 (16.3)	20 (17.5)	13 (14.8)
**Household Characteristics**			
Children in house	43 (21.3)	16 (14.0)	27 (30.7)
Receive SNAP	63 (31.2)	41 (36.0)	22 (25.0)
Receive WIC	10 (5.0)	5 (4.4)	5 (5.7)
Use food pantry	52 (25.7)	41 (36.0)	11 (12.5)
	**Mean (SD)**	**Mean (SD)**	**Mean (SD)**
Household size	2.5 (±1.5)	2.3 (±1.6)	2.7 (±1.3)
Monthly SNAP benefit ($)	203.9 (±119.3)	201.7± (133.2)	208.4 (±88.8)

NOTES:

* Some sample sizes are small due to missing information. SD = standard deviation.

**Table 2:** Identified Needs and Interests of Entire Sample (N = 202) and Comparisons of Clients from the Two Free Health Clinics

	Total Sample	DDN (n=114)	HTD (n=88)
	*N (%)	*N (%)	*N (%)
**Transportation**			
Personal vehicle	94 (46.5)	46 (40.4)	48 (54.6)
Bus	13 (6.4)	13 (11.4)	0 (0.0)
Ride from friend/family	32 (15.8)	8 (7.0)	24 (27.3)
Taxi/Car service	0 (0.0)	0 (0.0)	0 (0.0)
Bike	3 (1.5)	3 (2.6)	(0.0)
Other mode	26 (12.9)	24 (21.1)	2 (2.3)
**Interest in Nutrition Support Option and Education**			
Receive local produce	165 (81.7)	90 (79.0)	75 (85.2)
Receive recipes	143 (70.8)	69 (60.5)	74 (84.1)
Test tasting/cooking class	112 (55.5)	70 (61.4)	42 (47.7)
Home garden support	80 (39.6)	37 (32.5)	43 (48.9)
Grocery store tour with nutritionist	78 (38.6)	40 (35.1)	38 (43.2)
Home visit by nutritionist	53 (26.2)	26 (22.8)	27 (30.7)
**Interest in Food Plan for Health**			
Overall health	75 (37.1)	39 (34.2)	36 (40.9)
Weight management	63 (31.2)	30 (26.3)	33 (37.5)
Diabetes	55 (27.2)	24 (21.1)	31 (35.2)
Blood pressure	63 (31.2)	36 (31.6)	27 (30.7)
Heart disease	53 (26.2)	31 (27.2)	22 (25.0)
**Interest in Physical Activity Plan for Health**			
Overall health	75 (37.1)	38 (33.3)	37 (42.1)
Weight management	65 (32.2)	32 (28.1)	33 (37.5)
Diabetes	48 (23.8)	20 (17.5)	28 (31.8)
Blood pressure	60 (29.7)	33 (29.0)	27 (30.7)
Heart disease	49 (24.3)	27 (23.7)	22 (25.0)
**Interest in Nutrition and Health Online Modalities:**			
Interested in nutrition education through social media	61 (30.2)	32 (28.1)	29 (33.0)
Interested in nutrition education through online instructional videos	65 (32.2)	27 (23.7)	38 (43.2)

NOTES:

* All reported frequencies are an affirmative response to the category or question.
